# Assessing the relationship of recommended diarrhea case management practices with the nutritional status of children aged between 0 to 59.9 months.

**DOI:** 10.12688/healthopenres.13366.1

**Published:** 2023-11-29

**Authors:** Asif Khaliq, Abdul Rehman Shah Syed, River Holmes-Stahlman, Muhammad Fawad Tahir, Shamshad Karatela, Zohra S. Lassi

**Affiliations:** 1 School of Public Health & Social Work, Queensland University of Technology, Brisbane, Queensland, 4059, Australia; 2 Dow University of Health Sciences, Karachi, Sindh, 75510, Pakistan; 3 School of Biomedical Sciences, Queensland University of Technology, Brisbane, Queensland, 4059, Australia; 4 Department of Medicine and Surgery,, HBS Medical and Dental College, Islamabad, Pakistan; 5 School of Pharmacy, The University of Queensland, Saint Lucia, Queensland, 4072, Australia; 6 Institute of Tropical Health and Medicine (AITHM), James Cook University, Townsville City, Queensland, 4811, Australia; 7 School of Public Health, Faculty of Health and Medical Sciences., The University of Adelaide, Adelaide, South Australia, 5005, Australia; 8 Robinson Research Institute, Faculty of Health and Medical Sciences, The University of Adelaide, Adelaide, South Australia, 5005, Australia

**Keywords:** Diarrhoea, Practices, Paediatrics, Nutrition, Children

## Abstract

**Background:**

Paediatric diarrhoea and malnutrition have a bidirectional relationship, which in turn augments the presence of each other. The diarrhoeal diseases in children can be prevented by using *oral rehydration solution* (ORS) and *oral zinc sulfate*. The relationship between paediatric diarrhoea treatment guidelines with the nutritional status is not yet investigated, and this study primarily aims to examine the relationship of recommended diarrhoea case management practices with paediatric undernutrition (wasting, stunting and underweight) among children aged between 0 to 59 months.

**Methods:**

Data from *Pakistan Demographic & Health Surveys* (PDHS) conducted in 2012–2013 and 2017–2018 were used in this study to investigate the relationship between paediatric diarrhoea treatment adherence with the various forms of paediatric undernutrition. Data from children with complaints of acute watery non-dysenteric diarrhoea was used in this study, whereas data from those children presented with complaints of either dysentery, and/or severe dehydration, and/or incomplete anthropometry were excluded. Children were classified as complete adherent, partial adherent and non-adherent based on ORS and oral zinc sulfate consumption. The relationship of diarrhoea case management practices with different types of nutritional status was assessed using a binomial logistic regression method.

**Results:**

The malnutrition in children with acute non-dysenteric diarrhoea is 54.2% in 2012–2013, which decreased to 48.2% in the succeeding survey of 2017–2018. Only 6.2% (1% in 2012–2013 ~ 10% in 2017–2018) children of Pakistan received appropriate treatment for managing their diarrhoea. However, no relationship between the diarrhoea case management and paediatric undernutrition was observed.

**Conclusion:**

This study found did not demonstrate a significant connection of diarrhoea case management strategies with paediatric undernutrition. This highlights the complexity of addressing both diarrhoea and malnutrition in children. Future research should emphasize the identification and resolution of the multifactorial factors, which contribute to paediatric undernutrition, integrating both diarrhoea management and nutritional improvement strategies.

## Introduction

Diarrhoea is the second most common cause of death in children below five years of age ^1, 2^. It causes approximately 500,000 deaths in a year among children, accounting for almost 11.11% of child death per year ^3, 4^. In young children, the presence of at least three episodes of watery or loose stool within a 24-hour’s time is indicative of diarrhoea ^1, 5^. Diarrhoea in children is commonly associated with dehydration owing to severe fluid and electrolyte loss ^5, 6^. Dehydration in diarrhoea can be fatal if this is not treated in a timely manner ^4, 5^. However, timely and judicious use of certain straightforward, economical and affordable interventions helps to avert the morbidities, mortalities, and complications associated with paediatric diarrhoea ^7, 8^.

In children, most of the adversities associated with diarrhoeal disease can be effectively managed and prevented by following the “ *Integrated Management of Childhood Illnesses (IMCI)*” guidelines for diarrhoea management. This guideline is an outcome of joint efforts both the *World Health Organisation (WHO)* and *United Nations Children’s Fund (UNICEF)* ^9, 10^. This guideline gives a structured treatment plan, including the use of Oral Rehydration Solution (ORS), oral zinc, and adequate use of food & fluid for all children with acute watery non-dysenteric diarrhoea ^10, 11^. ORS is a cornerstone for replenishing the fluid and electrolyte loss associated with diarrhoeal diseases ^11, 12^. The ORS contains a mixture of various types of electrolytes with glucose. These electrolytes and glucose have the ability to replenish the fluid and electrolyte loss associated with diarrhoea ^9, 11, 12^. However, oral zinc helps to reduce the overall frequency and severity of paediatric diarrhoea ^5, 12, 13^. In conjunction with ORS, Zinc has been identified as a useful therapy for children older than six months with malnutrition ^8, 9^. The morbidity and mortality associated with paediatric diarrhoea can be overcome by following the recommendations prescribed in the IMCI guidelines ^13^

The morbidity and complications associated with diarrhoeal diseases are more prevalent in those countries with a high burden of malnutrition ^4, 14^. Many studies reported a synergistic and bidirectional relationship between paediatric diarrhoea and malnutrition ^15, 16^. Among South Asian countries, Pakistan shares a high burden of diarrhoea and malnutrition among children under five years old ^17, 18^. Malnutrition and poor diarrhoea case management practices are chiefly responsible for more than half of the deaths of children with diarrhoea ^13, 17, 18^. The findings of our previous study depicted poor case management practices for managing diarrhoeal disease in children aged below five years ^13^. However, the relationship of paediatric diarrhoea case management practices with nutritional status is not yet investigated. Owing to this reason, this study examined the relationship of recommended diarrhea case management practices with the nutritional status of children aged between 0 to 59 months using the datasets of *Pakistan Demographic & Health Surveys (PDHS)*.

## Methods

**Patient and Public involvement:** This study used data of neonates, infants and young children aged between 0 to 59 months from the datasets of *Pakistan Demographic & Health Surveys (PDHS)* conducted in 2012–2013 and 2017–2018. Our previous study showed poor maternal compliance for treating acute watery non-dysenteric paediatric diarrhoea ^13^. This finding derives the research question of our current study, which aim to investigate the relationship of paediatric nutritional status with diarrhoea case management practices using PDHS datasets.

 The data in each PDHS was collected by the officials of Demographic & Health Surveys (DHS), which restrict the authors to involve the study participants in the design, measure of outcomes, and study recruitment. However, the Key Finding Report (KFR) of each PDHS described detailed information about the design, data collection method, sample size, sampling methods, and various methods for the assessment of outcome.

**Study setting:** This is a community-based study in which datasets of the last two PDHS waves held in the years 2012–2013 and 2017–2018 were used for assessing the relationship of paediatric diarrhoea case management practices with their nutritional status. The PDHS is a national representative dataset that targets women of reproductive age for community-based data collection. The data in each PDHS was collected by trained and qualified data collectors with adequate training in participant contact, consent, communication, data collection, data management, and data measurement. The data collection in each PDHS was carried out by means of validated questionnaires, which were scrutinized and approved by the *Institutional review board of ICF, Pakistan Health Research Council (PHRC)*, and *National Bioethics Committee (NBEC), Pakistan* ^19^.

**Study design:** In this cross-sectional study, data pooling of 2012–2013 and 2017–2018 PDHS was performed to examine the nutritional profile of diarrhoea-positive infants and children of Pakistan. The data pooling aims to improve the sample size and statistical power of the study. Thus, enhancing the precision and validity between the exposure and outcome variables ^20^.

**Figure 1.**
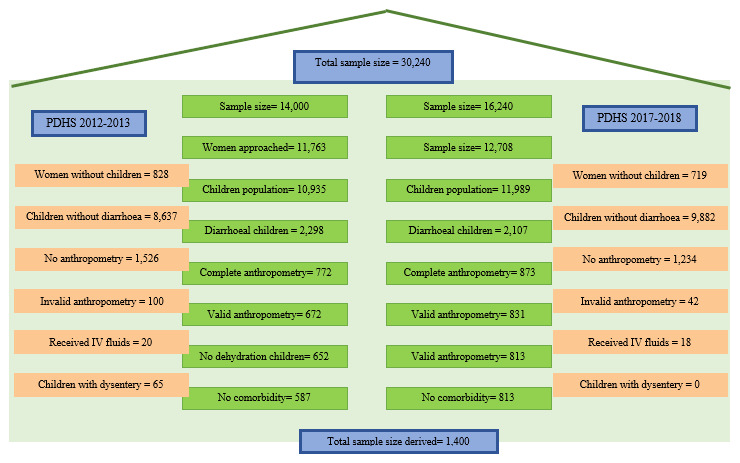
Sample size and sample selection.

**Study population:** This study targeted neonates, infants and children aged between 0 to five years, who experienced acute watery non-dysenteric diarrhoea. Acute watery non-dysentery diarrhoea refers to the passage of at least three watery stools in a day, having a maximum duration of fourteen days ^1, 5^. Additionally, children with complete anthropometry were also included. The complete anthropometry of a child includes data on the child's age in months, child sex, child weight in kilograms, child height or child length in centimeters and measuring position. However, data on children with dysentery (blood in stool), severe dehydration (those who received intravenous infusion for diarrhoea management), and anthropometric outliers (having HAZ score of ±6.00 S.D., WAZ score of -6.00 S.D. and +5.00 S.D. and WHZ score of ±5.00 S.D.) were excluded. Figure 1 presents an overview of the population selection of this study.

**Study size and sampling method:** The sample size for each PDHS was determined using the enumeration blocks (EBs), which typically encompassed approximately 200–300 households. The Pakistan Bureau of Statistics (PBS) and the most recent census records provided a list of these EBs in Pakistan. In 2012–2013, there were 500 EBs, and in 2017–2018, there were 580 EBs. Within each EB, the demographic surveillance team chose 28 households, resulting in sample sizes of 14,000 for 2012–2013 and 16,240 for 2017–2018, respectively. The total sample size for the PDHS was 30,240, with 24,471 (80.9%) women of reproductive age participating.

The selection of women for each PDHS was conducted through a multistage stratified cluster random sampling method. In 2012–2013, 11,763 women participated, while in 2017–2018, the number was 12,708. However, this study specifically focused on children under the age of five who presented with acute watery non-dysenteric diarrhea and no signs of dehydration. After excluding cases with comorbid conditions, anthropometric outliers, and other associated factors, the total number of children included in the study was 1,400 ( Figure 1)

## Variables

**Measurement of study outcomes:** Paediatric nutritional status was the primary outcome of this study, which was measured using anthropometric measurements. Anthropometric indices, which assess the nutritional status via z-score, were considered, such as height/length for age (HAZ/LAZ), weight for age (WAZ), and weight for height/length (WHZ/WLZ). However, Body Mass Index for Age (BAZ) was excluded because the BAZ is a poor indicator for measuring the paediatric nutritional status ^18^. In this study, each child’s nutritional status was examined in two hierarchical levels. At first, all the diarrhoeal children were bifurcated into two major groups: healthy and undernourished, based on the z-score values of each anthropometric index. Children with a z-score value ≤-2.00 S.D. for anthropometric indices were undernourished. Later, three subclasses of undernourished children were derived: Stunted, Underweight, and Wasted, again based on z-score. Children having z-score values below -2.00 S.D. for LAZ/HAZ were categorized as Stunted children. Similarly, any child with WAZ and WLZ/WHZ z-score values below -2.00 S.D. were considered underweight and wasted children.

**Measurement of study predictors:** The primary predictor variable of this study is the treatment strategies for the management of acute watery non-dysenteric diarrhoea. Use of ORS and oral zinc sulfate is the recommended treatment strategy for the management of acute watery non-dysenteric diarrhoea. Management of diarrhoeal episodes in children using either ORS and/or zinc sulfate represents appropriate practices or adherence to recommended treatment practices. However, use of home-based solution, antibiotics, antimotility medicines, rice starch, mint extract, herbal remedy, non-antibiotic medicines, and others for the management of diarrhoea in children are not recommended and their consumption either alone or in conjunction with other treatment modalities indicates non-adherence to the therapeutic guidelines. Further information about therapeutic adherence can be assessed from our previous paper ^13^.

**Study covariates:** For defining the relationship of paediatric diarrhoea case management practices with their nutritional status, we considered biological, household, and environmental factors. The biological factors include child age in months (0 to 11 months, 12 to 23 months, 24 to 35 months, 36 to 47 months, 48 to 59 months), child sex (male, female), and birth type (singlet, twin). Maternal education (no education, primary, secondary/higher), paternal education (no education, primary, secondary/higher), maternal occupation (working, full-time housewife), paternal occupation (working, unemployed), family size (1 to 7 members, 8 or more members), and the number of children below five years (one to two children, more than two children) were included as household factors. However, the environmental factors include the type of sanitation facility (improved facility, unimproved facility), type of drinking water available (improved water supply, unimproved water supply), and type of place of residence (rural, urban).

**Data sources and measurement:** A team of trained and experienced data collectors interviewed women of reproductive age across Pakistan using different sets of reliable and validated questionnaires in each PDHS. The data collectors used three different approaches for data collection: 1) Participant interview: The data collectors relied solely on participant responses for collecting information related to participant education, partner education, participant occupation, partner occupation, family size and birth type of the child. 2) Direct observation and participant interview: For certain variables, such as place of residence, housing infrastructure, type of toilet facility, and type of drinking water supply, the data collector relied on some standard guidelines, observations, and participant responses. 3) Measurement: For assessing children's health and nutrition status, the data collectors measured the weight and height of each child according to the criteria proposed by the WHO for measuring anthropometry. 

**Data access and cleaning methods:** This study used datasets of the last two PDHS waves conducted in the year 2012–2013 and 2017–2018. Both datasets contain around 1,000 variables of different types. This study considered socioeconomic and sociodemographic variables pertaining to paediatric diarrhoea and nutrition. Therefore, certain variables related to family planning, sexual health, malaria, hepatitis, and domestic violence were excluded. After excluding non-relevant data, descriptive analysis was performed, and these study variables provided information about the population of under five-year children in each study dataset. Similarly, data related to healthy children, children with dysentery, enteric fever, cold, cough, and flu were deleted from the datasets. Data of all the children with incomplete anthropometry and those with anthropometric outliers were also deleted.

Following data cleaning, descriptive statistics were performed to examine each variable's distribution of various categories. Additionally, missing data were also deleted. The percentage of missing data in PDHS 2012–2013 and 2017–2018 is 1.1% and 1.6%, respectively.

Certain variables related to diarrhoea treatment strategies were recorded and transformed into new variables called diarrhoea treatment adherence. The variable related to nutritional status in the actual dataset is numerical, which was first converted into three major categories of undernutrition based on the z-score values for each anthropometric index: Stunting, Wasting and Underweight. Subsequently, a computational analysis of nutrition-related factors was conducted to evaluate the prevalence of undernourishment in children with diarrhea.

Some variables, such as the type of toilet facility and source of drinking water, have more than ten categories. Extracting meaningful information from multiple categories is troublesome for the researcher and policymakers. Therefore, WHO has proposed a guideline for transforming various categories of toilet facilities and drinking water sources into Improved and Unimproved categories. 

**Statistical methods:** The data in this study was first analyzed descriptively. Following descriptive analysis, binomial regression was performed to examine the relationship of each recommended diarrhoea case management practice with the paediatric nutrition status. Unordered multinomial logistic regression was performed to investigate further the relationship of diarrhoea case management practices with each type of undernourishment ( Table 2– Table 4).

## Result

This study included data from 1,400 children from two datasets. Among 1,400 children, 587 (41.9%) were from 2012–2013, while the remaining 813 (58.1%) were from 2017–2018. Table 1 presents an overview of the different characteristics of the study sample.

**Table 1.** Characteristics of the study sample.

*Variable*	*2012–2013* *(n = 587)*	*2017–2018* *(n = 813)*	*Total* *(n = 1400)*
*Biological factors*			
***Child age (categorical)*** * 0 to 11 months* * 12 to 23 months* * 24 to 35 months* * 36 to 47 months* * 48 to 59 months*	138 (23.5%) 162 (27.6%) 136 (23.2%) 82 (14%) 69 (11.8%)	221 (27.2%) 220 (27.1%) 163 (20.0%) 126 (15.5%) 83 (10.2%)	359 (25.6%) 382 (27.3%) 299 (21.4%) 208 (14.9%) 152 (10.9%)
***Child sex*** * Male* * Female*	314 (53.5%) 273 (46.5%)	421 (51.8%) 392 (48.2%)	735 (52.5%) 665 (47.5%)
***Birth type*** * Singlet* * Twin/triplet*	579 (98.6%) 8 (1.4%)	797 (98%) 16 (2.0%)	1376 (98.3%) 24 (1.7%)
*Household factors*			
***Maternal formal *** ***education*** * None* * Primary* * Secondary* * Higher*	306 (52.1%) 89 (15.2%) 132 (22.5%) 60 (10.2%)	402 (49.4%) 120 (14.8%) 184 (22.6%) 107 (13.2%)	708 (50.6%) 209 (14.9%) 316 (22.6%) 167 (11.9%)
***Maternal work*** * Full time housewife* *Working mothers*	459 (78.2%) 127 (21.6%)	739 (90.9%) 74 (9.1%)	1199 (85.6%) 201 (14.4%)
***Paternal formal education*** * None* * Primary* * Secondary* * Higher*	177 (30.2%) 86 (14.7%) 207 (35.3%) 117 (19.9%)	214 (26.3%) 135 (16.6%) 275 (33.8%) 177 (21.7%)	391 (28.2%) 221 (15.9%) 482 (34.7%) 294 (21.2%)
***Paternal work*** * Employed* * EmployedUnemployed*	567 (96.6%) 20 (3.4%)	774 (96.6%) 27 (3.4%)	1341 (96.6%) 47 (3.4%)
***Family size*** * 1 to 7 members* * 8 or more members*	268 (45.7%) 319 (54.3%)	316 (38.9%) 497 (61.1%)	584 (41.7%) 816 (58.3%)
***Number of children below*** *** 5 years*** * 1 to 2 children* * More than 2 children*	389 (66.3%) 198 (33.7%)	490 (61.8%) 303 (38.2%)	879 (63.6%) 501 (36.3%)
*Environmental factors*			
***Type of place of residence*** * Urban* * Rural*	267 (45.5%) 320 (54.5%)	360 (44.3%) 453 (55.7%)	627 (44.8%) 773 (55.2%)
***Source of drinking water*** * Improved* * Unimproved*	509 (86.7%) 78 (13.3%)	689 (84.7%) 124 (15.3%)	1198 (85.6%) 202 (14.4%)
***Type of sanitation facility*** * Improved* * Unimproved*	434 (73.9%) 153 (26.1%)	614 (75.5%) 199 (24.5%)	1048 (74.9%) 352 (25.1%)
*Treatment factors*			
***Use of ORS*** * No* * Yes*	325 (55.4%) 262 (44.6%)	483 (59.4 %) 330 (40.6%)	808 (57.7%) 592 (42.3%)
***Use of zinc*** * No* * Yes*	574 (97.8%) 13 (2.2%)	706 (86.8%) 107 (13.2%)	1280 (91.4%) 120 (8.6%)
***Treatment adherence*** * No adherence* * Partial adherence* * Complete adherence*	318 (54.2%) 263 (44.8%) 6 (1.0%)	457 (56.2%) 275 (33.8%) 81 (10.0%)	775 (55.4%) 538 (38.4%) 87 (6.2%)

**Table 2.** Assessing the relationship between paediatric diarrhoea case management practices with stunting.

*Variable*	Unadjusted odds	Model 1	Model 2	Model 3
***Use of ORS*** * No* * Yes*	Ref 0.98 (0.79 to 1.22)	Ref 0.96 (0.75 to 1.22)	-	-
***Use of zinc*** * No* * Yes*	Ref 0.99 (0.67 to 1.45)	-	Ref *0.88 (0.57 to 1.36)*	-
***Treatment adherence*** * Non-adherence* * Partial adherence* * Complete adherence*	Ref 0.99 (0.79 to 1.24) 0.96 (0.61 to 1.52)	-	-	Ref 0.97 (0.75 to 1.25) 0.85 (0.51 to 1.43)
***Year of Survey*** * 2012–2013* * 2017–2018*	Ref 0.86 (0.69 to 1.06)	-	-	-
***Child age (categorical)*** * 0 to 11 months* * 12 to 23 months* * 24 to 35 months* * 36 to 47 months* * 48 to 59 months*	Ref 2.53 (1.81 to 3.52) * 5.84 (4.12 to 8.29) * 3.83 (2.62 to 5.59) * 3.39 (2.25 to 5.12) *	2.83 (1.99 to 4.04) * 7.25 (4.97 to 10.60) * 4.15 (2.76 to 6.23) * 4.10 (2.64 to 6.37) *	Ref 2.83 (1.98 to 4.03) * 7.26 (4.97 to 10.60) * 4.14 (2.76 to 6.22) * 4.12 (2.65 to 6.41) *	Ref 2.83 (1.99 to 4.05) * 7.28 (4.98 to 10.63) * 4.16 (2.77 to 6.24) * 4.12 (2.65 to 6.40) *
***Child sex*** * Male* * Female*	Ref 0.86 (0.69 to 1.07)	-	-	-
***Birth type*** * Singlet* * Twin/triplet*	Ref 1.71 (0.73 to 4.00)		-	-
***Maternal formal *** ***education*** * None* * Primary* * Secondary* * Higher*	Ref 0.69 (0.50 to 0.94) * 0.43 (0.32 to 0.57) * 0.22 (0.14 to 0.33) *	Ref 0.74 (0.52 to 1.04) 0.46 (0.33 to 0.63) * 0.24 (0.15 to 0.39) *	Ref 0.73 (0.52 to 1.04) 0.46 (0.33 to 0.63) * 0.24 (0.15 to 0.39) *	Ref 0.73 (0.52 to 1.04) 0.46 (0.33 to 0.63) * 0.24 (015 to 0.39) *
***Maternal work*** * Full time housewife* * Working mothers*	Ref 1.71 (1.26 to 2.32) *	Ref 1.51 (1.07 to 2.13) *	Ref 1.51 (1.07 to 2.12) *	Ref 1.51 (1.07 to 2.12) *
***Paternal formal education*** * None* * Primary* * Secondary* * Higher*	Ref 0.55 (0.39 to 0.78) * 0.48 (0.37 to 0.64) * 0.32 (0.23 to 0.44) *	-		
***Paternal work*** * Unemployed* * Employed*	Ref 0.88 (0.48 to 1.60)			
***Family size*** * 1 to 7 members* * 8 or more members*	Ref 1.08 (0.86 to 1.34)			
***Number of children below *** ***5 years*** * 1 to 2 children* * More than 2 children*	Ref 1.45 (1.07 to 1.96) *	Ref 1.46 (1.05 to 2.03) *	Ref 1.45 (1.04 to 2.02) *	Ref 1.51 (1.07 to 2.12) *
***Type of place of residence*** * Urban* * Rural*	Ref 1.02 (0.82 to 1.27)	-	-	
***Source of drinking water*** * Unimproved* * Improved*	Ref 0.78 (0.57 to 1.06)			
***Type of sanitation facility*** * Unimproved* * Improved*	Ref 0.53 (0.41 to 0.68) *	Ref 0.69 (0.51 to 0.92) *	Ref 0.69 (0.51 to 0.92) *	Ref 0.69 (0.51 to 0.92) *

Model 1 = The odds of stunting were adjusted with ORS use during the diarrhoeal episodes, child age, formal maternal education, maternal occupation, children under five years, and type of toilet facilities. Model 2 = The odds of stunting were adjusted with zinc use during the diarrhoeal episodes, child age, formal maternal education, maternal occupation, children below five years, and type of toilet facilities. Model 3 = The odds of stunting were adjusted with treatment adherence during the diarrhoeal episodes, child age, formal maternal education, maternal occupation, children below five years, and type of toilet facilities.

**Table 3.** Assessing the relationship between paediatric diarrhoea case management practices with underweight.

*Variable*	Unadjusted odds	Model 1	Model 2	Model 3
***Use of ORS*** *No* *Yes*	Ref 0.95 (0.75 to 1.21) *	Ref 1.02 (0.80 to 1.32)	-	
***Use of zinc*** *No* *Yes*	Ref 0.73 (0.47 to 1.14)	-	Ref 0.78 (0.49 to 1.24)	
***Treatment adherence*** *Non-adherence* *Partial adherence* *Complete adherence*	Ref 0.91 (0.71 to 1.16) 0.83 (0.50 to 1.39)			**Ref** 0.95 (0.73 to 1.23) 0.98 (0.57 to 1.66)
***Year of Survey*** *2012-2012* *2017-2018*	Ref 0.83 (0.65 to 1.05)			
***Child age (categorical)*** *0 to 11 months* *12 to 23 months* *24 to 35 months* *36 to 47 months* *48 to 59 months*	Ref 0.94 (0.67 to 1.32) 1.34 (0.95 to 1.89) 1.51 (1.04 to 2.20) * 1.25 (0.82 to 1.91) *			
***Child sex*** *Male* *Female*	Ref 0.90 (0.71 to 1.14)			
***Birth type*** *Singlet* *Twin/triplet*	Ref 2.22 (0.98 to 5.01)			
***Maternal formal education*** *None* *Primary* *Secondary* *Higher*	Ref 0.72 (0.51 to 1.00) * 0.40 (0.29 to 0.55) * 0.21 (0.12 to 0.36) *	Ref 0.71 (0.49 to 1.01) 0.49 (0.35 to 0.70) * 0.25 (0.14 to 0.42) *	Ref 0.70 (0.49 to 1.01) 0.49 (0.35 to 0.69) * 0.25 (0.14 to 0.42) *	Ref 0.71 (0.49 to 1.01) 0.50 (0.35 to 0.70) * 0.25 (0.15 to 0.43) *
***Maternal work*** *Full time housewife* *Working mothers*	Ref 1.67 (1.22 to 2.28) *	Ref 1.62 (1.16 to 2.26) *	Ref 1.62 (1.15 to 2.25) *	Ref 1.61 (1.15 to 2.26) *
***Paternal formal education*** *None* *Primary* *Secondary* *Higher*	Ref 0.79 (0.56 to 1.13) 0.55 (0.41 to 0.74) * 0.45 (0.32 to 0.65) *			
***Paternal work*** *Unemployed* *Employed*	Ref 0.46 (0.25 to 0.83) *			
***Family size*** *1 to 7 members* *8 or more members*	Ref 1.10 (0.87 to 1.39)			
***Number of children below *** ***5 years*** *1 to 2 children* *More than 2 children*	Ref 1.42 (1.03 to 1.96) *			
***Type of place of residence*** *Urban* *Rural*	Ref 0.94 (0.74 to 1.18) *			
***Source of drinking water*** *Unimproved* *Improved*	Ref 0.82 (0.59 to 1.14) *			
***Type of sanitation facility*** *Unimproved* *Improved*	Ref 0.54 (0.42 to 0.70) *	Ref 0.70 (0.53 to 0.94) *	Ref 0.71 (0.53 to 0.95) *	Ref 0.71 (0.53 to 0.94) *

Model, 1 = The odds of being underweight were adjusted with ORS use during the diarrhoeal episodes, formal maternal education, maternal occupation, and type of toilet facilities. Model 2 = The odds of being underweight were adjusted with zinc use during the diarrhoeal episodes, formal maternal education, maternal occupation, and type of toilet facilities. Model 3 = The odds of being underweight were adjusted with treatment adherence during the diarrhoeal episodes, maternal formal education, maternal occupation, and type of toilet facilities.

**Table 4.** Assessing the relationship between paediatric diarrhoea case management practices with wasting.

*Variable*	Unadjusted odds	Model 1	Model 2	Model 3
***Use of ORS*** *No* *Yes*	Ref 1.09 (0.77 to 1.53)	Ref 1.17 (0.82 to 1.67)		
***Use of zinc*** *No* *Yes*	Ref 0.81 (0.42 to 1.56)		Ref 1.13 (0.57 to 2.23)	
***Treatment adherence*** *Non-adherence* *Partial adherence* *Complete adherence*	Ref 1.05 (0.74 to 1.50) 0.96 (0.46 to 1.99)			Ref 1.08 (0.75 to 1.53) 1.46 (0.68 to 3.15)
***Year of Survey*** *2012–2012* *2017–2018*	Ref 0.63 (0.45 to 0.89) *	Ref 0.60 (0.42 to 0.85) *	Ref 0.59 (0.41 to 0.85) *	Ref 0.58 (0.40 to 0.84) *
***Child age (categorical)*** *0 to 11 months* *12 to 23 months* *24 to 35 months* *36 to 47 months* *48 to 59 months*	Ref 0.69 (0.46 to 1.04) 0.34 (0.20 to 0.58) * 0.31 (0.17 to 0.59) * 0.26 (0.12 to 0.56) *	Ref 0.62 (0.41 to 0.95) * 0.29 (0.16 to 0.50) * 0.30 (0.16 to 0.57) * 0.24 (0.11 to 0.53) *	Ref 0.63 (0.42 to 0.97) * 0.29 (0.17 to 0.59) * 0.30 (0.16 to 0.58) * 0.24 (0.11 to 0.53) *	Ref 0.63 (0.41 to 0.96) * 0.29 (0.16 to 0.50) * 0.30 (0.16 to 0.57) * 0.24 (0.11 to 0.52) *
***Child sex*** *Male* *Female*	Ref 1.13 (0.85 to 1.59)			
***Birth type*** *Singlet* *Twin/triplet*	Ref 0.76 (0.17 to 3.30)			
***Maternal formal education*** *None* *Primary* *Secondary* *Higher*	Ref 0.59 (0.34 to 1.00) 0.44 (0.26 to 0.72) * 0.62 (0.35 to 1.10)	Ref 0.51 (0.29 to 0.91) * 0.43 (0.26 to 0.72) * 0.61 (0.34 to 1.10)	Ref 0.51 (0.29 to 0.91) * 0.44 (0.27 to 0.74) * 0.61 (0.34 to 1.10)	Ref 0.51 (0.29 to 0.90) * 0.44 (0.26 to 0.73) * 0.61 (0.34 to 1.10)
***Maternal work*** *Full time housewife* *Working mothers*	Ref 1.08 (0.67 to 1.73)			
***Paternal formal education*** *None* *Primary* *Secondary* *Higher*	Ref 0.90 (0.53 to 1.54) 0.84 (0.55 to 1.30) 0.98 (0.60 to 1.58)			
***Paternal work*** *Unemployed* *Employed*	Ref 1.80 (0.82 to 3.94)			
***Family size*** *1 to 7 members* *8 or more members*	Ref 1.00 (0.71 to 1.41)			
***Number of children below *** ***5 years*** *1 to 2 children* *More than 2 children*	Ref 1.87 (1.23 to 2.83) *			
***Type of place of residence*** *Urban* *Rural*	Ref 0.90 (0.64 to 1.26)			
***Source of drinking water*** *Unimproved* *Improved*	Ref 0.93 (0.58 to 1.50)			
***Type of sanitation facility*** *Unimproved* *Improved*	Ref 0.71 (0.49 to 1.02)			

1 = The odds of wasting were adjusted with treatment adherence during the diarrhoeal episodes, year of survey, child age and formal maternal education. 2 = The odds of stunting were adjusted with treatment adherence during the diarrhoeal episodes, year of survey, child age and formal maternal education. 3 = The odds of being underweight were adjusted with treatment adherence during the diarrhoeal episodes, year of survey, child age and maternal formal education.

Across two survey periods, the study documented a significant improvement in the management practices for acute watery non-dysenteric diarrhea. In Pakistan, merely 6.2% of children under the age of five receive appropriate treatment for managing acute watery non-dysenteric diarrhoea. However, full compliance with the prescribed case management guidelines was only 1% (n=6) in 2012–2013, but it rose to 10% (n=81) during the 2017–2018 survey. The increase in complete adherence among diarrhoeal children was attributed to an increased intake of zinc sulfate in 2017–2018 (13%), compared to the formal survey of 2012–2013 (2.2%). Conversely, the 2017–2018 survey showed decreased ORS intake compared to the formal survey of 2012–2013 (i.e., 44.6% in 2012–2013 ~ 40.6% in 2017–2018). (see Table 1)

### Prevalence and Trend of malnutrition and its various types among diarrhoeal children of Pakistan

**Figure 2.**
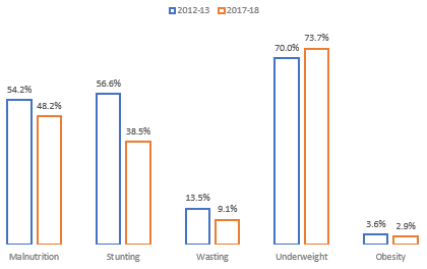
Prevalence and Trend of malnutrition and its various types among diarrhoeal children of Pakistan.

The 2017–2018 survey showed a significant decline in the prevalence of paediatric malnutrition and its various types compared with the former survey of 2012–2013. Between the two survey periods, there is a sharp decline of 6% in the paediatric malnutrition. In 2012–2013, more than half of children, i.e., 54.2% were malnourished, while the percentage of malnourished children decreased to 48.2% in the 2017–2018 survey. Among malnourished children, more than 95% of diarrhoeal children were undernourished, i.e., stunted, wasted, or underweight. However, the percentage of overnourished children, i.e., overweight and/or obese, was below 5% ( Figure 2).

***Association of paediatric diarrhoea treatment adherence with stunting:*** No association was observed between diarrhoea treatment adherence with paediatric stunting. Furthermore, using ORS and zinc sulfate showed no association with stunting among diarrhoeal children.

 Compared to children below 12 months, children aged between 24 and 35.9 months have more than seven times higher odds of stunting. Similarly, children of working mothers and a household with more than two children below five years showed increased stunting. Conversely, children of mothers with education at least secondary level reported significantly lower odds of stunting than children of mothers without education. Similarly, children living with improved sanitation facilities showed 31% (95% CI: 8% to 49%) lower odds of stunting than those living in households with unimproved sanitation facilities ( Table 2).

***Association of paediatric diarrhoea treatment adherence with underweight:*** A non-significant association exists between ORS use and underweight. Similarly, the use of zinc among diarrhoeal children was not associated with paediatric underweight. Moreover, no association exists between diarrhoea treatment adherence with the paediatric underweight.

 Our study reported significantly lower odds of being underweight among children of mothers having at least secondary level education than children of uneducated mothers. Similarly, children living in households with improved sanitation facilities showed significantly lower odds of being underweight than those with unimproved sanitation facilities. Conversely, children of working mothers showed 1.5 folds higher odds of being underweight compared with children of non-working mothers ( Table 3)

***Association of paediatric diarrhoea treatment adherence with wasting:*** This study reported no association of paediatric wasting with diarrhoea treatment adherence. Similarly, no association of paediatric wasting with ORS and/or zinc was reported.

This study reported higher odds of paediatric wasting in children over one year compared to young children aged below one year (0 to 11 months). However, lower odds of paediatric wasting were observed in children of mothers having either a primary or secondary level of education compared to non-educated mothers. Similarly, the current survey of 2017–2018 reported 41% (15% to 59%) lower odds of paediatric wasting compared to the former survey of 2012–2013 ( Table 4)

## Discussion

This study is among the few studies examining the relationship between paediatric diarrhoea case management practices and their nutritional status. In children under five years of age, acute watery non-dysenteric diarrhoea can be managed using ORS and zinc sulfate ^10, 13^. Apart from electrolyte imbalance and dehydration, malnutrition is an immediate complication of diarrhoeal diseases ^16^. However, the findings of this study did not show a significant association between paediatric diarrhoea case management practices with any forms of paediatric undernutrition: stunting, wasting, or underweight. A possible reason for the lack of association between diarrhoea treatment adherence with various forms of paediatric undernutrition might be low adherence to ORS and zinc for managing acute watery non-dysenteric diarrhoea ^13^. However, this study reported a significant increase in adherence to the diarrhoea case management practices in the 2017–2018 survey compared to the former survey of 2012–2013 (1% in 2012–2013 ~ 10% in 2017–2018). This significant increase in treatment adherence was chiefly attributed to the increased intake of zinc sulfate in 2017–2018 compared to the previous survey of 2012–2013 (2.2% in 2012–2013 ~ 13.2% in 2017–2018) ^13^. An increase in zinc consumption has reduced paediatric wasting to approximately 40% in 2017–2018 compared to the 2012–2013 survey. A case-control study conducted in Karachi showed an around four-fold higher prevalence of zinc deficiency among underweight children having a z-score value of ≤-2.00 S.D, compared to normal-weight children ^21^. Similarly, a randomized controlled trial by Makonenn *et al.*, conducted in hospital settings of Lesotho, showed beneficial effects of zinc micronutrient supplementation against malnutrition and various preventable illnesses, such as diarrhoea, pneumonia, fever, and skin ^22^. The protective role of zinc sulfate against the vicious cycle of malnutrition was supported by other researchers ^23^. Thus, using zinc among diarrhoeal children can improve the health and nutrition of children under five years.

This study reported that less than half of children received ORS for managing the symptoms of acute watery non-dysenteric diarrhea across two survey periods. This aligns with our previous study, which reported approximately 40% ORS adherence use among children suffering from diarrhoea ^13^. However, using ORS in paediatric diarrhoea can reduce over 90 per cent of deaths in children ^24^. Similarly, in India, adherence to ORS use was observed in around one-third to half of the children ^25^. Even in African countries, such as Nigeria, around 38% of children under five years use ORS to manage acute watery non-dysenteric diarrhoea ^26^. Like ORS, oral zinc in children with diarrhoea reduces diarrhoea-associated mortality by 23% ^24^. However, this study reported low consumption of zinc sulfate for managing paediatric diarrhoea. The low consumption of zinc sulfate for managing paediatric diarrhoea might be attributed to nausea and vomiting associated with zinc use. Many studies reported nausea and vomiting among children receiving high doses of zinc ^27^. A multicenter randomized control trial conducted in two African and Asian countries demonstrated low incidence zinc induced nausea and vomiting in children taking either 5 mg or 10 mg of oral zinc compared with children using 20 mg of oral zinc for their diarrhoea case management. Thus, low-dose oral zinc can prevent zinc-induced nausea and vomiting among children with diarrhoea ^28^. 

Our study reported malnutrition among half of the diarrhoeal children. Similarly, another study conducted among the general paediatric population reported the same prevalence of paediatric malnutrition in Pakistan. The escalated prevalence of malnutrition among paediatric populations of Pakistan reveals that children are malnourished not only because of the high burden of common preventable illnesses, such as diarrhoea, pneumonia, and malaria but a variety of other underlying reasons pertaining to socioeconomic and demographic features that also contribute to the high prevalence of malnutrition in children ^18^. This study showed significantly higher odds of stunting in children aged over one year. Different studies conducted in Asia and Africa also showed that an increase in the child's age is associated with an increased prevalence of stunting in children ^29^. A multicounty analysis by Checkley *et al.* (2008) reported that stunting in children is the consequence of diarrhoeal reinfection. Moreover, a history of recurrent and/or chronic diarrhoea indicates paediatric stunting ^30^. Thus, this study reflects that most children must have a history of recurrent and/or chronic diarrhoea.

This study reported significantly lower odds of all forms of undernutrition in children of educated mothers compared to children of non-educated mothers. Similarly, our other study also reported a protective role of maternal education against various types of paediatric nutritional disorders ^31^. Various studies conducted in the past also supported the protective role of maternal education in alleviating paediatric malnutrition ^32^. An improvement in maternal education reduces the risk of paediatric undernutrition by approximately 15%. Similarly, a study conducted in Bangladesh also reflected a lower risk of undernutrition among children of educated mothers than children of uneducated mothers ^33^. The low prevalence of malnutrition among children of educated mothers might be attributed to appropriate childcare, which include giving taking care of diet, hygiene, and associated environment. A study conducted in Ethiopia showed significantly better childcare practices among educated mothers than among uneducated mothers ^34^. Moreover, access to the media, such as television, radio, and newspaper, improves maternal knowledge and paediatric nutritional status, which educated mothers have access to ^35^. However, this study reported higher odds of paediatric undernutrition among children of working mothers, and this high prevalence of paediatric undernutrition among children of working mothers is in line with the findings of our previous study ^13^. Similarly, a systematic review by Hosen *et al.* (2023) showed an increased prevalence of paediatric undernutrition among children of working mothers ^36^. The high risk of undernutrition among children of working mothers could be due to insufficient time for childcare. In this regard, all the workplaces must provide a childcare facility for the mothers of preschool children, and this facility will allow working mothers to look after their pre-school children, even at their workplace.

### Strengths and limitations

This study is amongst the few studies which examined the relationship of diarrhoea case management practices with the nutritional status of diarrhoeal children using nationally representative datasets. There are various parameters that strengthen the internal validity of this study, such as the nationally representative sample size derived from the record of the last census, the multistage stratified sampling method, and the use of validated questionnaires for data collection. Experienced and trained data collectors and anthropometrists having three to four months training were involved in the data collection of each PDHS. Similarly, the data used in this study was of excellent quality because of the presence of less than 10% missing values and outliers. Furthermore, the data was normally distributed because the skewness and kurtosis of each continuous variable of this study were under the normal range of ±1.0 S.D ^37^.

 Despite having numerous strengths, the cross-sectional design of this study weakens the relationship between diarrhoea case management practices and paediatric nutritional status. The datasets used in this study measured the prevalence of paediatric diarrhoea retrospectively. A fourteen-day retrospective window was used for assessing the prevalence of paediatric diarrhoea and treatment practices employed for managing paediatric diarrhoea. Therefore, this study could not provide information about the current health status of children, i.e., how many children had diarrhoea at the time of data collection and how many children have recovered from the diarrhoeal ^38^. Additionally, this study failed to investigate the frequency, duration, and repetition of diarrhoeal disease in children below five years of age. In this way, the existing data of this study cannot confirm the exact prevalence of acute watery non-dysenteric diarrhoea. There might be chances that the existing data contains a proportion of chronic and/or persistent diarrhoea cases having complaints of watery diarrhoea over 14 days and 30 days, respectively.

Similarly, the data collectors recruited in this study had only focused on the use of different treatment strategies that can be used for the management of paediatric diarrhoea. However, they did not enquire about the frequency and duration of different treatment strategies. Hence, this study was not able to examine duration specific relationship of recommended diarrhoea case management practices. Similarly, the information related to diarrhoeal disease and its management strategies solely depended on the caregiver's response. There might be chances of reporting biases. The shortcomings of this study encourage the need for prospective interventional research, which can specifically focus on children with acute non-dysenteric diarrhoea having diarrhoea complaints not more than 14 days. Additionally, the effect of recommended diarrhoea case management practices should be examined according to the recommended duration of treatment.

## Conclusion

This study did not show a significant association between diarrhoea case management practices and paediatric undernutrition (wasting, stunting, and underweight). This suggests that improving adherence to treatment guidelines alone has no influence on the nutrition profile of children suffering from diarrhoea. This study reported a protective role of oral zinc towards the prevention of acute forms of malnutrition, i.e., wasting. However, the finding of this study did not provide robust evidence regarding the role of oral zinc in preventing paediatric undernutrition among diarrhoeal children. Future research should focus on identifying and addressing several effective interventions that could encompass diarrhoea management and nutritional improvement strategies among paediatric populations.

## Ethics and consent

### Institutional review board statement

The access to the use of this study data was granted by the data archivist of the *Demographic and Health Surveys (DHS) Program* for the project entitled ***“Diarrhoea treatment adherence and its effects on paediatric nutrition status”*** on 27 ^th^ February 2023.

### Informed consent statement

In this study, the research team received de-identified data, i.e., it does not contain information in which a participant can either be identified or traced by any means. Due to this reason, this study does not involve a statement for informed consent.

## Data Availability

Data used in this study are from the Standard DHS survey dataset of Pakistan, 2012–2013 and 2017–2018, Standard DHS surveys, available from the Demographic and Health Survey (DHS) website www.dhsprogram.com. Access to the dataset requires registration and is granted only for legitimate research purposes. A guide for how to apply for dataset access is available at: https://dhsprogram.com/data/Access-Instructions.cfm.
