# Supplementary material for: Assessing the relationship of recommended diarrhea case management practices with the nutritional status of children aged between 0 to 59.9 months
Source: Health Open Res. 2023 Nov 29;5:34. [Version 1] doi: 10.12688/healthopenres.13366.1 (PMC11638907; doi:10.12688/healthopenres.13366.1)
Supplement: Peer review history

# Reviewer report: Reviewer response for version 1

**DOI:** 10.21956/healthopenres.14451.r28240

**Published:** 2024-06-10

**Authors:** Erni Astutik

**Affiliations:** 1 Universitas Airlangga, Surabaya, East Java, Indonesia

Thank you for the opportunity to review this manuscript. This manuscript is essential issue in public health problem. I have some comments to improve the quality of manuscript.

  

1. The author needs to add more information on why it is important to conduct research on the management of diarrhea in relation to nutritional status. The information provided by the author is still not strong enough in introduction.
2. I think authors need to be consistent when using term, such as children aged 0 to five years or children aged 0 to 59 months. I think these terms have different meaning.
3. DHS has many datasets; authors need to add information which dataset used to analyze the data
4. Authors need to explain clearly about main independent variable (case management practices). This information is not clear, in the results, authors divided case management practice into: using ORS, using zink, and Treatment adherence. However, authors did not explain them in the methods section
5. I think authors need to be consistent using the term of case management practice. Because it is different in the title, method, and results.
6. Figure 1, please add the sample size used in this study in each phase.
7. Can the authors provide references when categorizing family size? Because I have found different ways of categorizing the family size variable in several journals
8. In the method section, subheading “data source and measurement”, authors need to add the references about information. Because the paragraph gives the impression that the author is using primary data, whereas the data used is actually secondary data.
9. DHS uses complex sampling. Have you accounted for the use of complex sampling design in your data analysis? Please explain and add this information in the methods section under data analysis.
10. Authors have not explained all variables in the method section. For example, type of sanitation facility, use of ORS, and use of zinc, and treatment adherence. Please explain the data management in the method section.
11. Why did authors add year variable in the table 2,3, and 4. Because, authors did not explain the variable earlier. Could you add explanation?
12. In discussion section, authors can add the general finding based on the aim of the study, then compare with other studies and explain the possible mechanism. Because, authors explained directly about ORS and zinc sulfate. It was confusing.
13. I think, authors need to sharpen the discussion why no relationship between the diarrhoea case management and paediatric undernutrition was observed
14. Please add the policy implication based on your results

Is the work clearly and accurately presented and does it cite the current literature?

Yes

If applicable, is the statistical analysis and its interpretation appropriate?

Yes

Are all the source data underlying the results available to ensure full reproducibility?

Yes

Is the study design appropriate and is the work technically sound?

Yes

Are the conclusions drawn adequately supported by the results?

Yes

Are sufficient details of methods and analysis provided to allow replication by others?

Yes

Reviewer Expertise:

Public Health and Epidemiology

I confirm that I have read this submission and believe that I have an appropriate level of expertise to confirm that it is of an acceptable scientific standard.

# Reviewer report: Reviewer response for version 1

**DOI:** 10.21956/healthopenres.14451.r28242

**Published:** 2024-06-10

**Authors:** Muhammad Ilyas

**Affiliations:** 1 Ministry of Public Health Qatar, Doha, Qatar

The article provides a valuable examination of the relationship between recommended diarrhea case management practices and the nutritional status of young children in the low resource settings like Pakistan. The study design and methodology are sound, and the authors have presented their findings in a clear and well-organized manner. The data analysis appears to be rigorous and the conclusions drawn are supported by the results.

 However, there are a few areas where the authors could strengthen the manuscript. The introduction could be expanded to provide more context around the importance of this research topic and to clearly articulate the study objectives.

 1-The introduction is generally well-written but could benefit from some minor language adjustments. While the paper discusses diarrhea and its complications, it lacks detailed information on the incidence, prevalence, morbidities, and mortalities associated with diarrhea. The introduction does not clearly state whether the provided status information is global or specific to Pakistan. Additionally, the introduction does not provide any background information or status on malnutrition, which is the primary outcome of the study.

 This is an important omission, given that the nutritional status of children is the primary outcome variable of the study. Providing only general statements about diarrhea and malnutrition, without grounding them in concrete epidemiological evidence, weakens the introduction.

 To strengthen this section, the authors should incorporate more specific, well-sourced data on the burden of diarrhea and malnutrition, clarifying whether the figures are global, national, or regional in scope. This contextual information would help establish the significance and relevance of the research question.

 2- Under the conclusion, The first statement is confusing as " The study either found or did not demonstrate".  Please make correction. 

 Introduction: 

 1-(Made the language corrections as below, the bold and underline words need revision.): 

 Diarrhoea is the second most common cause of death in children below five years of age ^1^ ^,^ ^2^ . It causes approximately 500,000 deaths **in a year** annually among children

 2- economical and affordable interventions helps to avert the morbidities, complications and mortalities associated with paediatric diarrhoea ^7^

 (changed the order complications and mortalities in the sentence)  

 Methods:

 1- In the second paragraph, the second part of this statement is not needed **which restrict the authors to involve the study participants in the design, measure of outcomes, and study recruitment.**

**Study population**

 2-Additionally, children with complete anthropometry were **also** included. Also or ONLY The revised statement shall be Additionally, children with complete anthropometry were **only** included.

**Data access and cleaning methods.**

 Since this is national level secondary dataset, It would include many variables. Instead of describing the PDHS, it would be better to report on why and how the list of variables in this analysis were extracted from the data source. 

 3-Additionally, missing data were also deleted. The percentage of missing data in PDHS 2012–2013 and 2017–2018 is 1.1% and 1.6%, respectively. 

 The authors should explicitly state the percentage or proportion of missing values for the key variables analyzed here. Reporting PDHS overall missing values are not much relevant here. Similarly, if reporting, then the implications on this analysis shall also be clearly mentioned. 

**Table 1. Characteristics of the study sample**

**1- **treatment adherence is not defined in the data section before Table 1

 2-Labele for ***Paternal work *** is not correct. Check labels of both categories. Cat 2 is wrong. 

 Table 2. Assessing the relationship between paediatric diarrhoea case management practices with stunting

 1-The decimal is missing in 015 for maternal education, Higher category, in model 3. It shall be 0.15 instead.

 Discussion; 

 This statement is incomplete, after skin...(it is not clear that what skin conditions etc)

  Similarly, a randomized controlled trial by Makonenn *et al.*, conducted in hospital settings of Lesotho, showed beneficial effects of zinc micronutrient supplementation against malnutrition and various preventable illnesses, such as diarrhoea, pneumonia, fever, and skin

 Finally the Figure 2 is not visually very clear and blurs. A good quality image would be more readable.

Is the work clearly and accurately presented and does it cite the current literature?

Yes

If applicable, is the statistical analysis and its interpretation appropriate?

Yes

Are all the source data underlying the results available to ensure full reproducibility?

Yes

Is the study design appropriate and is the work technically sound?

Yes

Are the conclusions drawn adequately supported by the results?

Yes

Are sufficient details of methods and analysis provided to allow replication by others?

Yes

Reviewer Expertise:

Maternal and child health, Demographic surveillance, Quality control and health services performance

I confirm that I have read this submission and believe that I have an appropriate level of expertise to confirm that it is of an acceptable scientific standard.

# Reviewer report: Reviewer response for version 1

**DOI:** 10.21956/healthopenres.14451.r28247

**Published:** 2024-05-13

**Authors:** Shafaq Taseen

**Affiliations:** 1 The Aga Khan University Hospital, Karachi, Pakistan

The manuscript provides valuable insights into the relationship between diarrhea case management practices and pediatrics undernutrition in Pakistan. It is well-written, structured, and supported by robust methodology. The findings contribute to our understanding of the complexities surrounding pediatric health and nutrition in low-resource settings. Addressing the identified limitations through future research endeavors will further enhance the knowledge base and inform evidence-based interventions.

Is the work clearly and accurately presented and does it cite the current literature?

Yes

If applicable, is the statistical analysis and its interpretation appropriate?

Yes

Are all the source data underlying the results available to ensure full reproducibility?

Yes

Is the study design appropriate and is the work technically sound?

Yes

Are the conclusions drawn adequately supported by the results?

Yes

Are sufficient details of methods and analysis provided to allow replication by others?

Yes

Reviewer Expertise:

Pediatric Health, Infectious Diseases, Public Health

I confirm that I have read this submission and believe that I have an appropriate level of expertise to confirm that it is of an acceptable scientific standard.
